# Supplementary material for: Identification of photoperiod-induced specific miRNAs in the adrenal glands of Sunite sheep (Ovis aries)
Source: Front Vet Sci. 2022 Jul 22;9:888207. doi: 10.3389/fvets.2022.888207 (PMC9354845; doi:10.3389/fvets.2022.888207)
Supplement: Supplementary file 1 [file Data_Sheet_1.ZIP › Supplementary file/miRDeep2 file.zip!/miRDeep2 file/Know_Structure/SP42A2/oar-mir-125b.pdf]

miRBase precursor : oar-mir-125b
Total read count : 257330
oar-miR-125b read count : 256397
remaining reads : 933

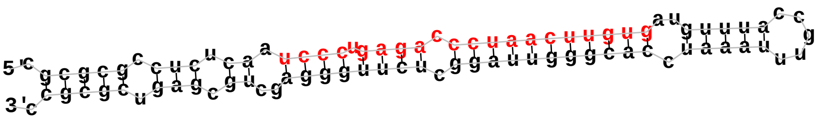

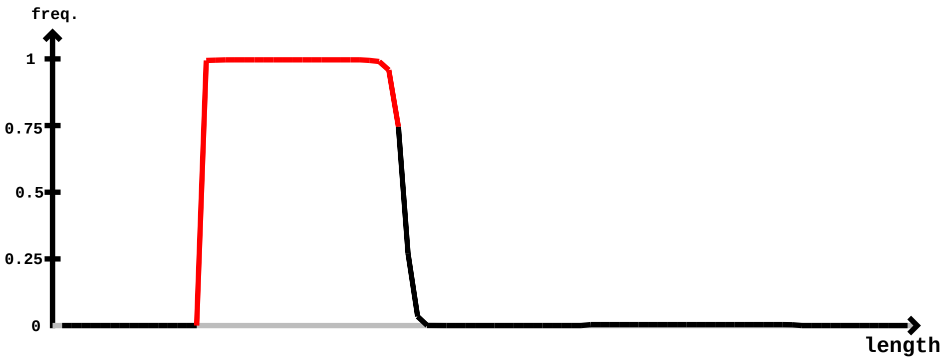

oar-miR-125b

```
5'- cgcgcgccucucaaucccugagacccuaacuugugauguuuaccguuuaaauccacggguuaggcucuugggagcugcgagucgcgcc   -3'    exp
    .(((((.(((.((.((((.((((.(((((((((((..(((((.....))))).))))))))))).))))))))..)).))).))))).    reads     mm        sample
    ............aaucccugagacccuaacuug.......................................................    1         0         seq
    ............aaucccugagacccuaacuugug.....................................................    1         0         seq
    ............aaucccugagacccuaacuuguga....................................................    1         0         seq
    .............Gucccugagacccuaacuugu......................................................    1         1         seq
    .............aucccugagacccuaacuugug.....................................................    4         0         seq
    .............Gucccugagacccuaacuuguga....................................................    1         1         seq
    .............aucccugagacccuaacuuguga....................................................    1         0         seq
    .............aucccugagacccuaacuugugau...................................................    1         0         seq
    ..............ucccugagGcccuaacu.........................................................    6         1         seq
    ..............ucccugagacccuaacu.........................................................    464       0         seq
    ..............ucccugagacccuGacu.........................................................    2         1         seq
    ..............ucccugagaUccuaacu.........................................................    2         1         seq
    ..............ucccAgagacccuaacu.........................................................    1         1         seq
    ..............ucccugagacccuaacC.........................................................    78        1         seq
    ..............Acccugagacccuaacu.........................................................    3         1         seq
    ..............uUccugagacccuaacu.........................................................    1         1         seq
    ..............ucccugagacccuaacCu........................................................    12        1         seq
    ..............ucccugagacccuaacuu........................................................    1047      0         seq
    ..............ucccugagacccuaacuug.......................................................    8206      0         seq
    ..............ucccugagacccuaacuugu......................................................    54721     0         seq
    ..............ucccugagacccuaacuugug.....................................................    121828    0         seq
    ..............ucccugagacccuaacuuguga....................................................    60700     0         seq
    ..............ucccugagacccuaacuugugaG...................................................    2228      1         seq
    ..............ucccugagacccuaacuugugau...................................................    6387      0         seq
    ..............ucccugagacccuaacuugugaug..................................................    49        0         seq
    ..............ucccugagacccuaacuugugaGg..................................................    11        1         seq
    ..............ucccugagacccuaacuugugaugu.................................................    40        0         seq
    ..............ucccugagacccuaacuugugaGgu.................................................    2         1         seq
    ..............ucccugagacccuaacuugugauguu................................................    1         0         seq
    ...............cccugagacccuaacuug.......................................................    5         0         seq
    ...............cccugagacccuaacuugu......................................................    13        0         seq
    ...............cccugagacccuaacuugug.....................................................    26        0         seq
    ...............cccugagacccuaacuuguga....................................................    233       0         seq
    ...............cccugagacccuaacuugugau...................................................    7         0         seq
    ...............cccugagacccuaacuugugaG...................................................    8         1         seq
```

oar-miR-125b

```
cgcgcgccucucaaucccugagacccuaacuugugauguuuaccguuuaaauccacggguuaggcucuugggagcugcgagucgcgcc
................ccugagacUcuaacuug.......................................................    1     1    seq
................ccugagacccuaacuug.......................................................    10    0    seq
................ccugagacccuaacuugu......................................................    78    0    seq
................ccugagacccuaacuugug.....................................................    136   0    seq
................ccugagacccuaacuuguga....................................................    61    0    seq
................ccugagacccuaacuugugaG...................................................    2     1    seq
................ccugagacccuaacuugugau...................................................    8     0    seq
.................cugagacccuaacuugug.....................................................    3     0    seq
.................cugagacccuaacuuguga....................................................    3     0    seq
.................cugagacccuaacuugugau...................................................    1     0    seq
.................cugagacccuaacuugugaG...................................................    1     1    seq
..................ugagacccuaacuugug.....................................................    1     0    seq
.....................................................cacggguuaggcucuuggga...............    1     0    seq
.....................................................cacggguuaggcucuugggagc.............    2     0    seq
.....................................................cacggguuaggcucuugggagcu............    3     0    seq
......................................................acggguuaggcucuuggg................    5     0    seq
......................................................acggguuaggcucuuggga...............    2     0    seq
......................................................acggguuaggcucuugggag..............    10    0    seq
......................................................acggguuaggcucuugggagc.............    105   0    seq
......................................................acggguuaggcucuugggagcu............    797   0    seq
......................................................acggguuaggcucuugggagcug...........    6     0    seq
.......................................................cggguuaggcucuugggagcu............    1     0    seq
........................................................ggguuaggcucuugggagcu............    1     0    seq
```
